# Cerebral malaria induces electrophysiological and neurochemical impairment in mice retinal tissue: possible effect on glutathione and glutamatergic system

**DOI:** 10.1186/s12936-017-2083-6

**Published:** 2017-11-02

**Authors:** Karen R. H. M. Oliveira, Nayara Kauffmann, Luana K. R. Leão, Adelaide C. F. Passos, Fernando A. F. Rocha, Anderson M. Herculano, José L. M. do Nascimento

**Affiliations:** 1 Laboratório de Neurofarmacologia Experimental, Instituto de Ciências Biológicas, Universidade Federal do Pará, R. Augusto Corrêa, 01, Belém, PA 66075-110 Brazil; 2 Laboratório de Neuroquímica Molecular e Celular Instituto de Ciências Biológicas, Universidade Federal do Pará, Belém, Pará Brazil; 3 Laboratório de Neurofisiologia Eduardo Oswaldo Cruz, Instituto de Ciências Biológicas, Universidade Federal do Pará, Belém, Pará Brazil

**Keywords:** Cerebral malaria, Glutathione, Glutamate uptake, Electroretinogram and cone photoreceptor response

## Abstract

**Background:**

Cerebral malaria (CM) is a severe complication resulting from *Plasmodium falciparum* infection. This condition has usually been associated with cognitive, behavioural and motor dysfunctions, being the retinopathy the most serious consequence resulting from the disease. The pathophysiological mechanisms underlying this complication remain incompletely understood. Several experimental models of CM have already been developed in order to clarify those mechanisms related to this syndrome. In this context, the present work has been performed to investigate which possible electrophysiological and neurochemistry alterations could be involved in the CM pathology.

**Methods:**

Experimental CM was induced in *Plasmodium berghei*-infected male and female C57Bl/6 mice. The survival and neurological symptoms of CM were registered. Brains and retina were assayed for TNF levels and NOS2 expression. Electroretinography measurements were recorded to assessed a- and b-wave amplitudes and neurochemicals changes were evaluated by determination of glutamate and glutathione levels by HPLC.

**Results:**

Susceptible C57Bl/6 mice infected with ≈ 10^6^ parasitized red blood cells (*P. berghei* ANKA strain), showed a low parasitaemia, with evident clinical signs as: respiratory failure, ataxia, hemiplegia, and coma followed by animal death. In parallel to the clinical characterization of CM, the retinal electrophysiological analysis showed an intense decrease of a- and-b-wave amplitude associated to cone photoreceptor response only at the 7 days post-infection. Neurochemical results demonstrated that the disease led to a decrease in the glutathione levels with 2 days post inoculation. It was also demonstrated that the increase in the glutathione levels during the infection was followed by the increase in the ^3^H-glutamate uptake rate (4 and 7 days post-infection), suggesting that CM condition causes an up-regulation of the transporters systems. Furthermore, these findings also highlighted that the electrophysiological and neurochemical alterations occurs in a manner independent on the establishment of an inflammatory response, once tumour necrosis factor levels and inducible nitric oxide synthase expression were altered only in the cerebral tissue but not in the retina.

**Conclusions:**

In summary, these findings indicate for the first time that CM induces neurochemical and electrophysiological impairment in the mice retinal tissue, in a TNF-independent manner.

## Background

Cerebral malaria (CM) is one of the major complication of *Plasmodium falciparum* infection and even with optimal anti-malarial treatment, more than 25% of cases result in death or cognitive and visual impairment [1–3]. A pathophysiological symptom associated to CM is the convulsive status epilepticus, characterized by constant seizures crisis [4]. Clinical evaluations performed in children infected with *P. falciparum* have described epileptic seizures associated with CM. In addition, it was showed that development of latter epilepsy in adults is a recognized sequel related with the development of CM during infancy or adolescence [5, 6]. Previous studies have already described that CM results from a complex and exacerbated inflammatory response of the infected host, which can lead to endothelial activation and disruption of blood–brain-barrier (BBB) with consequent neurodegenerative events [7–10]. It is well characterized that the development of CM is closely associated with the sequestration of infected red blood cells (iRBCs) and/or leukocytes in the cerebral microvascular endothelium leading to blood flow obstruction and decreased tissue perfusion, thereby compromising the function of distinct areas of the central nervous system (CNS), including the retinal tissue [11–15]. Although visual system represents a regular target of CM [14, 16], few studies described the neurochemicals mechanisms involved in retinal injury induced by CM. In fact, data from literature demonstrated that retinal tissue of patients with CM show an intense astrogliosis, astrocytes degeneration and microglia activation after longer periods of the disease onset [11, 12, 17]. However, it remains unclear the alterations in the retinal activity and neurotransmitters changes induced by this disease.

Retinal physiology is dependent on distinct neurotransmission systems and on the maintained redox status of the tissue [18–20]. In the retina, glutamatergic system represents the main excitatory pathway being responsible for the synaptic transmission between photoreceptor cells, bipolar cells and ganglion cells and its excessive action in different types of receptors may result in neuronal cell death by excitotoxicity [21]. Thereby, termination of glutamatergic neurotransmission is achieved by the removal of glutamate from the extracellular space by proteins transporters located in the plasmatic membrane of pre-synaptic terminals and surrounding glial cells [22, 23]. Several reports have already demonstrated the potential involvement of glutamate-mediated cytotoxicity in both acute and chronic neurodegenerative conditions [24, 25]. In the retina, it was characterized the presence of different transporters systems which include the Na^+^-dependent and Na^+^-independent glutamate transporters [26]. Added to this, previous studies have already showed that changes in the glutamate transport could be associated to electrophysiological response in retina and conditions of oxidative stress [27, 28].

In the CNS, including retinal tissue, the tripeptide glutathione (GSH) is the main antioxidant compound. GSH is formed by glutamate, cysteine and glycine residues and its production and release to synaptic cleft is essential for keeping the redox status in the tissue [29, 30]. In fact, it is well described that intracellular levels of GSH regulates survival and homeostasis of retinal tissue. Decrease of GSH content is associated with the induction of oxidative stress in retinal tissue and can evoke impairment of visual response [31–33]. The full-field electroretinogram (ERG) is the record of summed transient electrical responses from the entire retina elicited by a flash stimulus and is a widely used electrophysiologic test of retinal function [34]. The standard full-field ERG for clinical investigations was established by the International Society for Clinical Electrophysiology of Vision ISCEV [34]. The full-field ERG is essential in the diagnosis of numerous disorders as cone-rod and cone dystrophy [35], and diabetic retinopathy [36]. Furthermore, the use of ERG in combination with animal models are essentials to understand the physiological changes caused by diseases that affect the visual system.

The aim of the present study was to investigate how cones photoreceptors are affected by malarial retinopathy in mice with CM. In this way, the lack of studies demonstrating the neurochemicals events and retinal activity in the pathophysiology of CM incited the development of the present work which uses C57Bl/6 mice infected with *Plasmodium berghei* as an experimental model.

## Methods

### Ethics statement

All experimental procedures described had prior approval by the Animal Ethics Committee of the Federal University of Para (Protocol Number: 194-14/UFPA) and were conducted according to the Health Guide for the Care and Use of Laboratory Animals. All efforts were made to minimize animal suffering and to reduce the numbers of animals used. Drugs as ketamine and xylazine were used for anaesthetizing mice prior the experiments.

### Animals

Male and female C57BL/6 mice (weighing 20–22 g) 6- to 8-weeks old were obtained from Animal Care Facilities of the Institute of Biological Sciences, Federal University of Para (Para, Brazil). Animals were maintained in pathogen-free conditions, housed in groups of 10 mice per cage in a light-controlled room (12:12 h light–dark cycle) and temperature (23 ± 1 °C) with food and water ad libitum.

### *Plasmodium berghei* experimental infection

*Plasmodium berghei* strain ANKA were kindly provided by Evandro Chagas Institute (Para, Brazil) and maintained as frozen stocks in liquid nitrogen until the experiments. Mice were randomly divided into two groups: a non-infected control group (n = 8/experiment) and a *P. berghei*-infected group (n = 15/experiment). C57BL/6 mice were intraperitoneally (i.p.) injected with 10^6^ parasitized red blood cells (pRBCs) suspended in 0.1 mL phosphate-buffered saline (PBS) pH 7.0 [34]. Mice injected only with 0.1 mL PBS served as control group (uninfected mice). Survival and disease signs were checked daily in accordance with pre-defined humane endpoints. To evaluate the disease onset, mice were monitored twice a day for CM symptoms which consist of, at least, one of the following clinical signs of neurological involvement: poor reflexes, ataxia, limb paralysis and seizures. Behavioural changes, such as ataxia and seizures were used as humane endpoints to reduce the animal suffering. Terminal mice were anaesthetized with ketamine/xylazine (150–10 mg/kg) and killed by cervical dislocation. Parasitaemia (percentage of pRBCs) was assessed daily by microscopic counting and calculated as follows: [(number of pRBCs)/(total numbers of RBCs counted)] × 100 from Giemsa-stained thin smears obtained from tail blood.

### TNF ELISA

Concentrations of tumour necrosis factor (TNF) in cortical and retinal tissue were determined by specific enzyme-linked immune assay according to standard protocol provided by the company (Biolegend). All the samples were obtained from control and *P. berghei*-infected mice (on day 2, 4 and 7 post-infection) and the cellular supernatant was collected and stored at − 80 °C until the time of the assay. Plates were read at 450 nm and the quantification was achieved using standards curves obtained according to manufacturer’s guide.

### Electroretinography analysis

During different periods of infection (2, 4 and 7 days post infection) full-field electroretinography (ff*ERG*) was performed to assess retinal function in accordance to Harazny et al. [37]. In photopic conditions, mice were light-adapted for 10 min and stimulated with flashes of 10.2 cd s m^−2^ to obtain cone response. Control mice and *P. berghei*-infected mice were anaesthetized by injection of ketamine (100 mg/kg) and xylazine (6 mg/kg). Pupils were fully dilated with tropicamide (1%). Briefly, a silver disk electrode (model F-E6SHC-12, Grass Technologies, Warwick, USA) was placed in ear as ground, a silver ring electrode was placed on the cornea as active electrode and a subcutaneous steel needle electrode (model F-E3-48, Grass instruments, Warwick, USA) on the eyelid as reference electrode. After positioning the electrodes, the animals were placed in a Faraday cage and stimulated by visual stimulator (model PS33-PLUS, Grass Technologies, Warwick, USA). A xenon lamp (PS33-PLUS) was used to generate stimulus of white light, whose brightness was measured with digital luxmeter-photometer (Model LD-300). Intervals between stimulus was 4 s for photopic conditions with 10 ms of flashes duration. Light-evoked responses were amplified 50,000× with a AC amplifier (model P511, Grass Technologies, Warwick, USA), filtered between 0.3 and 300 Hz and digitalized with an analog–digital interface (National Instruments, Austin, TX). Data acquisition program used was Labview 3.0 (National Instruments, Austin, TX).

For each response have been issued 6–12 pulses, then the responses were based on the average response to all the white flashes presented. Amplitude (µV) was used as ERG parameters. a-wave amplitudes were measured from baseline to the a-wave trough and b-wave amplitudes were measured from the a-wave trough to the b-wave peak.

### Western-blotting analysis

Mice from uninfected and *P. berghei*-infected group were perfused with PBS for 2 min and cortical brain and retinal samples were quickly removed and frozen until use. Tissues were homogenized in buffer containing protease inhibitor mixture (Sigma) and after sonication, the homogenates were centrifuged at 16,000*g* and the supernatant was collected. Immunoblotting was performed with standard western blot principles. 20 µg of proteins were loaded onto reducing sodium dodecyl sulfate polyacrylamide gel electrophoresis (SDS-PAGE), and electrotransferred to a nitrocellulose membrane. The non-specific binding sites were blocked with 5% BSA in TBS-Tween for 60 min at room temperature. The blots were then incubated with NOS-2 primary antibody (1:200 in blocking solution) overnight at 4 °C. Antibody reactivity was detected using ECL-Plus chemiluminescence substrate (Amersham Pharmacia Biotech) and exposed to film (Sigma).

### GSH assay

Intracellular levels of glutathione (GSH) were determined by the method described by Anderson [38] with minor modifications in the enzymatic incubation time and concentration of PBS/EDTA solution. This procedure allows to determine, spectrophotometrically, the total levels of glutathione (GSH and GSSG) by the reduction of 5,5′-dithio-bis-2-nitrobenzoic acid (DTNB) in nitrobenzoic acid (TNB). In this reaction, the sample was incubated in two different groups: the first one was incubated only with DTNB to measure the levels of –SH group and in the second one was incubated with GSH reductase (that converts GSSG into GSH), NADPH and DTNB to measure the total levels of GSH produced by the reaction. The final result is expressed by the difference between these two groups as the total levels of glutathione.

After dissection, retinal tissue from control and *P. berghei*-infected groups (on day 2, 4 e 7 post infection) was resuspended in PBS/EDTA (1 mM), sonicated and centrifuged at 3000 rpm, during 10 min. The supernatant was separated and an aliquot was incubated in assay solution containing PBS/EDTA (1 mM); GSH reductase (100 μ/ml) and NADPH (4 mM). After 15 min, DTNB (5 mM) was added to the reaction and the amount of TNB was measure by spectrophotometry at 412 nm. Total GSH were expressed in percentage of the control.

### L-[^3^H]glutamate uptake assay in retinal explants

Retinal explants from control and *P. berghei*-infected groups were used to perform the glutamate uptake assay as described previously [26]. To evaluate glutamate uptake, retinal explants were washed four times with modified Hank’s balanced salt solution, which consists of (in mM): NaCl 128, KCl 4, MgCl_2_ 1, CaCl_2_ 2, glucose 12 and HEPES 20, adjusted to pH 7.4, and then incubated with [^3^H]glutamate (1 µCi/ml) during 10 min. All uptake assays were performed at room temperature (37 °C) in shaking. After [^3^H]glutamate time incubation, the uptake was stopped by rapidly rinsing the tissue three times with ice-cold Hank’s solution and then, tissue were lysed with TCA 5% and the intracellular glutamate content was determined by liquid scintillation spectroscopy. Results were expressed in % of control. Small aliquots were removed from each well for the determination o protein content by Bradford Method.

### Statistical analysis

Survival curves were compared using long-rank test. The normality test was performed using the Shapiro–Wilk test and the statistical difference between all groups was evaluated using one-way ANOVA followed by *Tukey* post-test. For comparisons of two groups, the Student’s *t* test (normally distributed) was used. Data are presented as mean ± SD and all experiments were repeated at least three times. Statistical analysis was carried out using GraphPad Prism Software and *p* values < 0.05 were considered significant.

## Results

### Development of experimental cerebral malaria (ECM) in C57Bl/6 mice infected with *Plasmodium berghei*

**Fig. 1:**
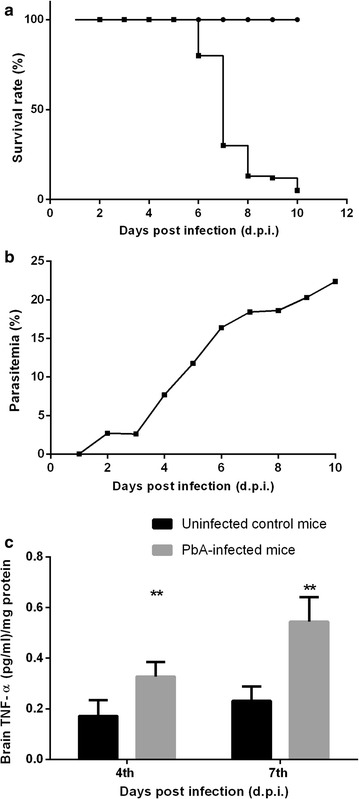
Survival rate (**a**), time course of parasitaemia (**b**) and brain TNF concentration (**c**) were assessed in C57Bl/6 mice infected with *Plasmodium berghei* ANKA (n = 15) and in uninfected control mice (n = 8). Mice were sacrificed upon the development of severe ECM symptoms. Data represented the mean ± SD and values significantly different by one-way ANOVA followed by Tukey post test: **p < 0.01 vs uninfected control mice. Experiments were performed three times with similar results

In Fig. 1, a set of results from different experimental procedures were conducted to characterize ECM in C57Bl/6 mice. Disease progression was assessed daily in individual mice by monitoring clinical symptoms, survival rate, peripheral blood parasitaemia and TNF immunological response. Infected mice developed typical ECM symptoms between 4 and 7 days post infection such as ataxia, hemiplegia, disorientation, respiratory dysfunction, multiple convulsions and died within 10 days post infection. Survival rate evaluation showed a progressive pattern of animals death into the *P. berghei*-infected group with ~ 100% of mortality in the first 10 days (Fig. 1a). Based in the behavioral symptoms and mortality pattern these animals were classified as cerebral malaria group. Consistent with earlier observations, other symptoms such as a rapid increase in parasitaemia (18% at 7 days post infection) were also observed in cerebral malaria animals (Fig. 1b). Ultimately, immunological analysis in cortical brain tissue of cerebral malaria animals demonstrated a significant increase on TNF levels at 4 and 7 days post infection when compared with uninfected mice (Fig. 1c). Taken together, these results confirm the development of cerebral malaria in those animals infected with *P. berghei.*

### Functional changes detected by ERG in *P. berghei*-infected mice are not associated with immunological activation

**Fig. 2:**
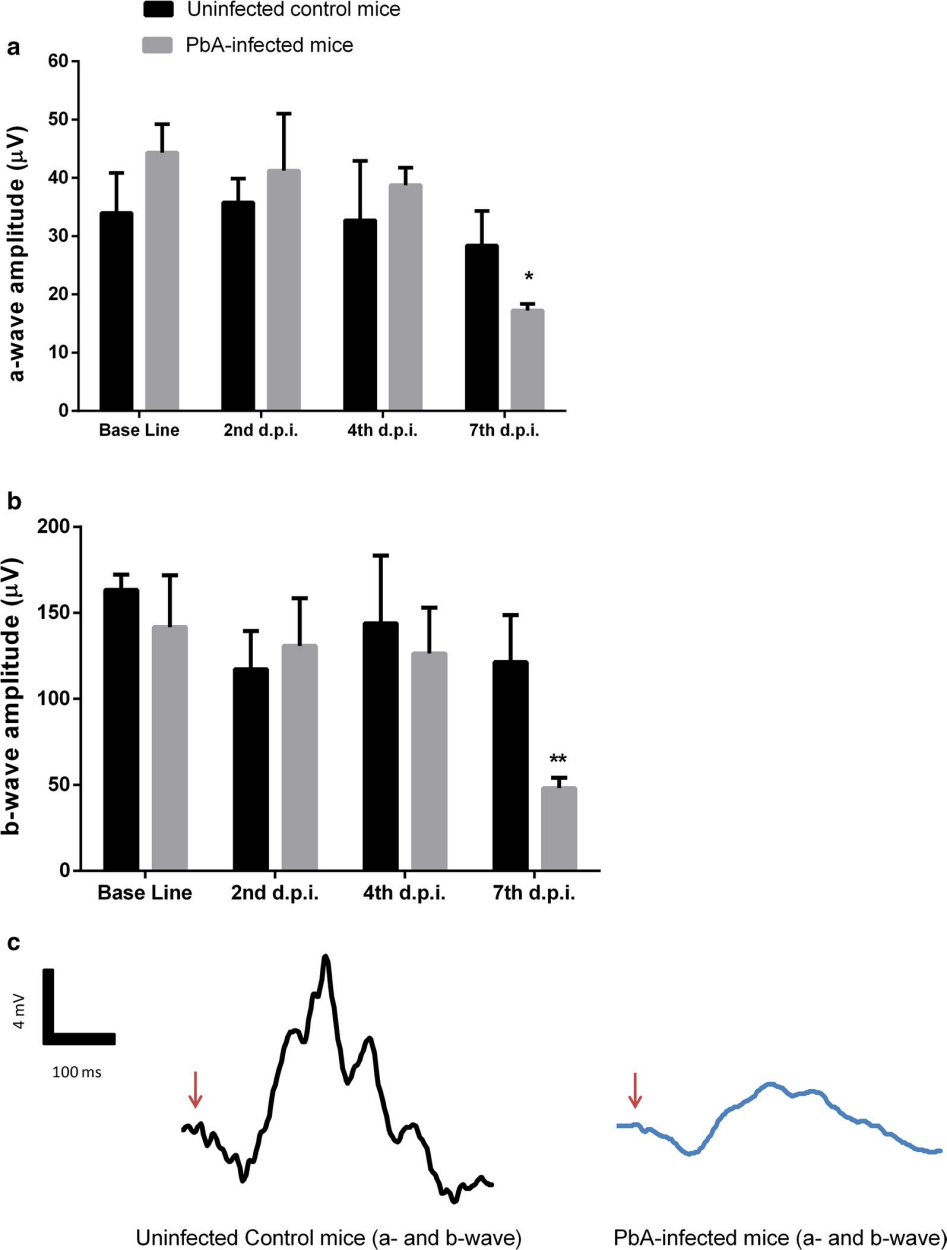
Electroretinography alterations of C57BL/6 mice at 2, 4 and 7 days post inoculation (d.p.i.) with *P. berghei* ANKA strain. **a** a-wave amplitude; **b** b-wave amplitude and **c** representative electroretinography record. Red arrow indicates the beginning of the stimulus. Data expressed by means of the groups. *p < 0.05 vs uninfected mice at 7 d.p.i.; **p < 0.01 vs uninfected mice at 7 d.p.i. (ANOVA and Tukey post test)

The development of ECM symptoms let us to evaluate whether retinal tissue of *P. berghei*-infected animals showed any alterations in the electrophysiological response during experimental disease. Retinal function was assessed by ERG under scotopic and photopic conditions in which scotopic ERG reflects rod function whereas photopic is related to cone cells. As demonstrated in Fig. 2, a significant reduction in photopic a- and b-waves was found in *P. berghei*-infected mice retina, suggesting a predominant cone photoreceptor function loss.

The electroretinographic analysis of *P. berghei*-infected mice demonstrated no significant changes in a- and b-wave amplitude associated to cone photopic response until 2 and 4 days post infection (Fig. 2a, b). However, a significant reduction in photopic a- and b-waves amplitudes (~ 12 and 83%, respectively) was found in *P. berghei*-infected mice retina at 7 days post infection (Fig. 2a, b). Representative waveforms elicited at 7 days post infection are shown in Fig. 2c.

**Fig. 3:**
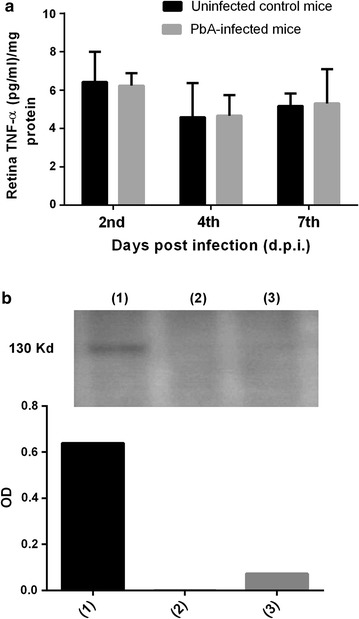
Retinal TNF levels (**a**) in C57BL/6 mice at 2, 4 and 7 days post inoculation (d.p.i.) with *P. berghei* ANKA strain and expression of NOS-2 enzyme (**b**) in the brain and retinal tissue of C57BL/6 mice on day 7 after inoculation with *P. berghei* ANKA strain. Arbitrary optical densitometry (OD) units from western blot of NOS2. Results were expressed as mean ± SD. (ANOVA; *post*-*test* Tukey–Kramer); (1) brains of infected animals; (2) retina of control animals; (3) retina of infected animals

In order to evaluate whether electroretinographical changes and inflammatory events in retinal tissue occurred simultaneously, TNF levels and NOS-2 expression was performed in retinal tissue of *P. berghei*-infected mice. The results demonstrated no significant changes in the retinal TNF levels at 2, 4 or 7 days post-infection (Fig. 3a). Moreover, while brain cortex of *P. berghei*-infected mice showed an intense expression of NOS-2, no enzymatic expression was observed in retinal tissue of *P. berghei*-infected mice at 7 day post infection (Fig. 3b).

### GSH levels and glutamate uptake in retinal tissue of *P. berghei*-infected mice

Considering that the electrophysiological impairment observed in *P. berghei*-infected mice seems not to be associated with the activation of an inflammatory pathway, it was evaluated whether CM evokes neurochemicals changes in retinal tissue. Firstly, as the retinal tissue is very susceptible to oxidative stress conditions, it was determined the concentration of GSH, the main antioxidant in the central nervous system, including the retina.

**Fig. 4:**
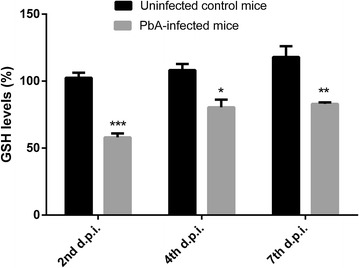
Intracellular GSH levels in the retinal tissue of C57BL/6 mice at 2, 4 and 7 days post inoculation (d.p.i.) with *P. berghei* ANKA strain. Datas were expressed by means of the groups. ***p < 0.0001 vs control at 2 d.p.i.; *p < 0.001 vs control at 4 d.p.i.; **p < 0.001 vs control at 7 d.p.i. (ANOVA, Tukey–Kramer post-test)

**Fig. 5:**
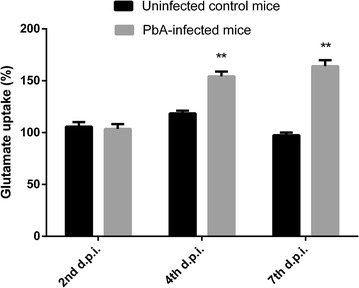
Glutamate uptake in retinal tissue of C57BL/6 mice at 2, 4 and 7 days post inoculation (d.p.i.) *P. berghei* ANKA strain. Data expressed by means of the groups. **p < 0.001 vs control (ANOVA, Tukey–Kramer post-test)

As shown in Fig. 4, at 4 days post infection, retinal tissue of *P. berghei*-infected mice presented a significant decrease in GSH amount (~ 24%) when compared with uninfected mice. Similar results were also observed at 7 days post-infection (Fig. 4). As glutamate is the principal neurotransmitter involved in oxidative stress conditions, our further experiments were performed to study whether functional glutamate uptake is altered by *P. berghei* infection. As shown in Fig. 5, glutamate net uptake was increased in retinal tissue at 4 and 7 days post-infection (~ 42 and ~ 61%, respectively), when compared with uninfected mice.

## Discussion

Cerebral malaria is a complex and multifactorial syndrome in which pathophysiology mechanisms underlying the CNS damage, including the retina are still not fully understood. Although murine CM model has provided important insights into the pathogenesis of this syndrome [39], neuropathological studies have paid little attention to the neurochemicals mechanisms involved in the course of the disease. In this way, the current study demonstrated, for the first time, that CM induced by *P. berghei* infection evokes significant neurochemical and electrophysiological changes in mice retinal tissue.

The retinopathy associated to CM has important features as retinal whitening, vessel discoloration, haemorrhages focus and reduced perfusion as a result from the mechanical obstruction due to pRBCs sequestration [14, 40]. Previous studies, using retinal wholemounts, describes the breakdown of blood–brain barrier (BBB), petechial haemorrhages and large edema in latter stages of mice experimental CM [41, 42]. Furthermore, it was also showed that the increased BBB permeability induced by *P. berghei*-infection leads to a microglia thickness and a redistribution of this cell toward the retina vasculature [43]. Increased c-fos expression evidencing an intense glial activation was also demonstrated in the retinal tissue of mice with CM, as well as, a highlighted astrogliosis and astrocytes degeneration [44, 45].

Noteworthy, the current work complements these findings showing that CM evokes functional changes in the retinal physiology which was evidenced by low photopic response (reducing a- and b-wave amplitude) observed in mice at 7 days post *P. berghei* infection. These results are in agreement with early observations which describe ophthalmologic dysfunctions in children with CM induced by *P. falciparum* infection [46, 47]. The tissue events responsible for the retinal electrophysiological impairment induced by CM are still not fully understood. However, some reports have pointed out that vessel obstruction and hypoxia generate by distinct diseases can induce visual dysfunction associated to altered response of cone photoreceptors [48–54]. Although the present work do not demonstrate a direct association between electrophysiological impairment and retinal hypoxia induced by CM, this hypothesis could be tested in posterior studies.

It was also observed that electroretinographical changes seems not to be associated to retinal inflammation since no increase on TNF levels or NOS-2 expression were observed in the retinal tissue at 7 days post-infection. The increased values of TNF and NOS-2 expression in the brain suggest that inflammatory events in the brain occur concomitantly with electrophysiological dysfunction described in retinal tissue of CM mice, but not with retinal TNF inflammation. Lou et al. [55] described that increased levels of TNF is an important biomarker of brain inflammation since its values are closely associated to inflammatory events in the brain such as haemorrhage events, plugging microvessels and upregulation of cellular adhesion molecules (CAMs). Besides TNF expression, several pro-inflammatory mediators such as IFN-γ, IL-1β and IL-6 contribute to the pathogenesis of CM in both humans and rodents, acting in part by increasing the expression of intercellular adhesion molecule-1 (ICAM-1) and vascular cell adhesion molecule-1 (VCAM-1) [56–59].

Also, previous reports have suggested that nitric oxide (NO), a product of the nitric oxide synthase enzymes (NOS), may also contribute to the pathogenesis of CM but its mechanism of action during the disease is controversial [60, 61]. Maneerat et al. [62] demonstrated a widespread expression of NOS-2 in different cells types in the brain during the acute phase of mice CM. Furthermore, overproduction of NO might disrupt the regulatory role of NO in CNS, leading to oxidative damage, disturbing signaling in the brain [63, 64].

Prior studies have suggested a role of oxidative stress produced both by host and parasite in the acute neurological condition development during the establishment of CM in mice [65, 66]. Further, previous reports have already showed that electrophysiological disruption in retinal tissue is accomplished to severe alterations on the redox status induced by different agents or disease conditions [67–70]. In the present study, it was demonstrated that, at 7 days post infection, CM induces an intense decrease in the GSH content, the main antioxidant in the CNS. Wright et al. [71] describes that GSH depletion is associated with electroretinogram alterations including decrease of a- and b-wave response in diabetic rats. The authors also describe that GSH depletion did not alter implicit time of response. It is well documented that a-wave photopic response are associated with cone photoreceptor response and that decrease of antioxidant as GSH can evoke toxicity of cone cells in the retina [71, 72]. Several factors can be associated to GSH depletion in the retinal tissue, including the generation of local oxidative stress conditions [73]. Studies reveal that oxidant damage in retinal tissue are closely associated to glutamatergic system alterations [74–76], including the hyperstimulation of NMDA glutamate receptors which evoke excitotoxicity in retinal cells [77–79]. This excitotoxic pathway culminates in ROS production and intense consumption of intracellular GSH. In addition, it is well established that expression and activation of NOS2 enzyme is a calcium-independent mechanism related with immunological stimulation of different tissues and is not necessarily associated with glutamatergic excitotoxicity.

Added to this, previous studies also describes that alterations on the glutamate transport represents an indirect marker of glutamatergic system dysregulation of in the CNS, including the retina tissue [80–82]. In the present work, it was also demonstrated a significant increase of glutamate uptake in the retinal tissue of mice with CM. The high activity of glutamate transporters indicates a dysregulation of glutamate levels in the synaptic cleft and a consequent change in the electrical response of retinal tissue [83, 84]. Extracellular glutamate concentration controls the activation of second order neurons in retinal tissue responsible for the appearance of b-wave in the electroretinogram [83, 84]. In this way, increased activity of glutamate transporters could be associated with intense uptake of glutamate in retinal and consequent decrease of b-wave amplitude observed in our results. Previous studies have already suggested a role of glutamate neurotransmitter in the CNS dysfunction found in CM. They demonstrated increased levels of glutamate release in the brain of C57Bl/6 mice infected with *P. berghei* ANKA strain, being this release closely associated with neurological and behavioural symptoms in CM [85].

Thus, for the first time. it was demonstrated that CM induces neurochemical and electrophysiological impairment in retinal tissue and suggesting that murine model of CM can represent an important tool for pre-clinical evaluation of substances with potential protector effect against the retinal dysfunction associated to this disease.

## Conclusions

The results presented herein show that cerebral malaria induces a decrease in the activity of cone photoreceptors in the latter stages of the disease. This decrease was also attributed to the neurochemical changes in the retinal tissue, such as the diminished levels of antioxidant glutathione and the elevated levels of glutamate neurotransmitter. Moreover, the present study demonstrated that these alterations occurred in a TNF independent manner.
